# Blind fish have cells that see light

**DOI:** 10.1098/rspb.2023.0981

**Published:** 2023-07-12

**Authors:** Inga A. Frøland Steindal, Yoshiyuki Yamamoto, David Whitmore

**Affiliations:** ^1^ Department of Marine Biology, James Cook University, Townsville, Queensland 4811, Australia; ^2^ Department of Developmental and Cell Biology, University College London, London, WC1E 7HU, UK; ^3^ Australian Institute of Tropical Health and Medicine, James Cook University, 1/14-88 McGregor Road, Cairns QLD 4870, Australia

**Keywords:** circadian, light biology, cavefish, cell culture, *Astyanax mexicanus*, biological clock

## Abstract

Most animals on earth have evolved under daily light–dark cycles and consequently possess a circadian clock which regulates much of their biology, from cellular processes to behaviour. There are however some animals that have invaded dark ecosystems and have adapted to an apparently arrhythmic environment. One such example is the Mexican blind cavefish *Astyanax mexicanus*, a species complex with over 30 different isolated cave types, including the founding surface river fish. These cavefish have evolved numerous fascinating adaptations to the dark, such as loss of eyes, reduced sleep phenotype and alterations in their clock and light biology. While cavefish are an excellent model for studying circadian adaptations to the dark, their rarity and long generational time makes many studies challenging. To overcome these limitations, we established embryonic cell cultures from cavefish strains and assessed their potential as tools for circadian and light experiments. Here, we show that despite originating from animals with no eyes, cavefish cells in culture are directly light responsive and show an endogenous circadian rhythm, albeit that light sensitivity is relatively reduced in cave strain cells. Expression patterns are similar to adult fish, making these cavefish cell lines a useful tool for further circadian and molecular studies.

## 1. Introduction

Being able to predict and anticipate the onset of day and night has been such a useful way of organizing biology that a time-keeping mechanism, a circadian clock, is ubiquitous in most organisms [1–5]. The circadian clock regulates a range of biological processes in animals, from basic cell biology to complex behaviours. In order for the clocks to work, they must be set to a local time, with the sun being the most potent resetting signal, known as a zeitgeber [6,–8]. However, there are organisms that have adapted to live in darkness without light as a zeitgeber, or any apparent entraining signals, such as cave animals. As far as we know, there are only two vertebrates (salamanders and teleosts) that have adapted to a cave environment. While there are only a handful of troglobitic salamanders, almost 300 species of cavefish have been described, with 150 of these species being described only in the last two decades [9]. The cavefishes are a polyphyletic group, with fish from 28 different families found in 36 different countries, but they share a range of common adaptations to cave animals (troglobites), including loss/reduction of eyes, loss of pigmentation and scales, alterations in metabolism, reduction in sleep, arrhythmic behaviours, as well as deregulation of all circadian and light biology [10–12]. Cavefishes originate from surface fishes that have been trapped in caves, thus cavefish descend from fish with a circadian clock. The time since cave invasion depends on the species of cavefish but varies from 10 000 years to maybe as much as 10 Myr [13–15].

Most cavefish are incredibly elusive and rare, they are hard to obtain and while some cavefish survive in aquaria, they do not reproduce. As a result, we therefore know very little about most species of cavefishes. Despite this, there are a few species that breed in captivity [16]. One of these species is the Mexican blind cavefish *Astyanax mexicanus*, which is the most researched of cave species. This species of tetra has over 30 cave morphs, but it also has the ancestral surface morph still swimming around in rivers in the El Albra region of Mexico [17]. Cave and surface *A. mexicanus* are interfertile, thus we can mate them in the laboratory, producing fertile F1 offspring. Such hybridization events also occur in the wild, such as in the Chica cave that experiences influx of surface fish during flooding events [18]. Having the founding species, as well as a range of isolated cave morphs, makes it an amazing tool for studying adaptations and the evolution to life in arrhythmic conditions.

All species of cavefish that have been explored to date are behaviourally arrhythmic in the wild, but most retain the ability to entrain to a light–dark (LD) cycle when housed in the laboratory [19]. There are, however, three species that have been reported to have no entrainable locomotor activity rhythm [20,21], with one of these species *Phreatichthys andruzzii* reported to have no circadian genes that oscillate in response to light [20]. These fish do, however, respond behaviourally to shading experiments and alter feeding behaviour in response to light, suggesting that some light-sensitive structures are retained [22,23]. Furthermore, cell lines created from *P. andruzzii* also show changes in ROS when exposed to light [24]. *A. mexicanus* cavefish on the other hand have retained a large opsin diversity and show both molecular and behavioural rhythms under artificial LD cycles in the laboratory [25–28]. However, the *A. mexicanus* cavefish morphs show a different expression pattern in key clock and light genes with a broader, delayed acrophase to that of surface morphs [19]. There are also differences between the cave morphs in terms of circadian expression patterns and transcriptomes, as well as a difference in direct light responses [19]. This suggests multiple independent mechanisms have evolved for loss of light and circadian biology. This hypothesis is further underpinned by genomic studies on other species of cavefishes that show mutations in a range of clock genes, suggesting that there is no particular clock or light-input gene that is under selective pressure across species [19,20,29–31].

*Astyanax mexicanus* is one of the few species of cavefish that breed in the laboratory. As opposed to conventional model species such as zebrafish, *A. mexicanus* has a long generational time (12 months) and spawning beaviours can be sporadic and unreliable. Furthermore, the *A. mexicanus* are 2-3 times larger than zebrafish, resulting in lower stocking densities, meaning that the same number of Mexican blind cavefish take up five times as much space as zebrafish. This in effect means that there is usually a big obstacle for experiments that require time series or have multiple experimental stages. Zebrafish cell lines have proven a very useful tool for circadian and light biology studies, and with cavefish having retained light responses in adult fish and embryos, we hypothesize that cell lines derived from these animals will retain similar properties. If this is the case, then such ‘cave’ cell lines could provide a very useful tool, not only for clock/light studies, but also for other evolutionary changes that occur at the cell/molecular level. We therefore decided to generate *A.mexicanus* cell cultures from cavefish and surface fish embryos and trial these as a circadian *in vitro* model.

Here we show that despite coming from a blind cavefish, embryonic cells in culture are still able to detect and respond to light. However, cell cultures from cave strains show significantly reduced sensitivity to light compared to embryonic surface cells. We also show that the four different populations of cave fish can all entrain to an LD cycle and show endogenous rhythmic expression patterns similar to adult fin clips and embryonic/larval fish. As our cell lines are typically generated from larval/embryonic stages, we have included additional, new data exploring light and clock function in two strains of cavefish larvae, as an extension to our previously published results in surface and Pachón embryonic stages. These results now allow for a full comparison of clock/light function in an *in vitro* scenario compared to the whole animal situation.

## 2. Results

### (a) Induction of clock genes depends on duration and intensity of light pulse

Cell lines and cell cultures are a ‘much-loved’ tool across molecular sciences. While mammalian cells have been somewhat difficult to work with in circadian biology as they need serum shock or other pharmacological manipulations to set the clock, zebrafish cells are directly light responsive, and as such cells in culture can entrain to an LD cycle [32]. This inherent light responsiveness is something we also believe is the case for other fish species with larger marine fish also being shown to be directly light responsive prior to visual structures forming [33]. Fish have a large genetic diversity of non-visual photopigments [34], with cavefish having 33 visual and non-visual photopigment encoding genes [25].

In order to determine the light responsiveness of the newly created cell lines, and to examine if there is a difference in sensitivity between the cave strains, we subjected the cells to three different intensities for either 5 or 10 min, at constant temperature. Cells were entrained for 4 days on a 12 : 12 LD cycle prior to the experiment, and ZT21 was chosen as the time of the light pulse, as previous phase response curve data in zebrafish has shown that this is the time of day that the zebrafish cells are robustly light responsive [35]. The light-responsive clock genes *cry1a* and *per2a*, as well as the light-sensitive DNA repair gene *cyclobutane pyrimidine dimer photolyase* (*CPD-Ph*), were selected as marker genes for light sensitivity, as these genes appear to be activated using different pathways [36–39].

**Figure 1.**
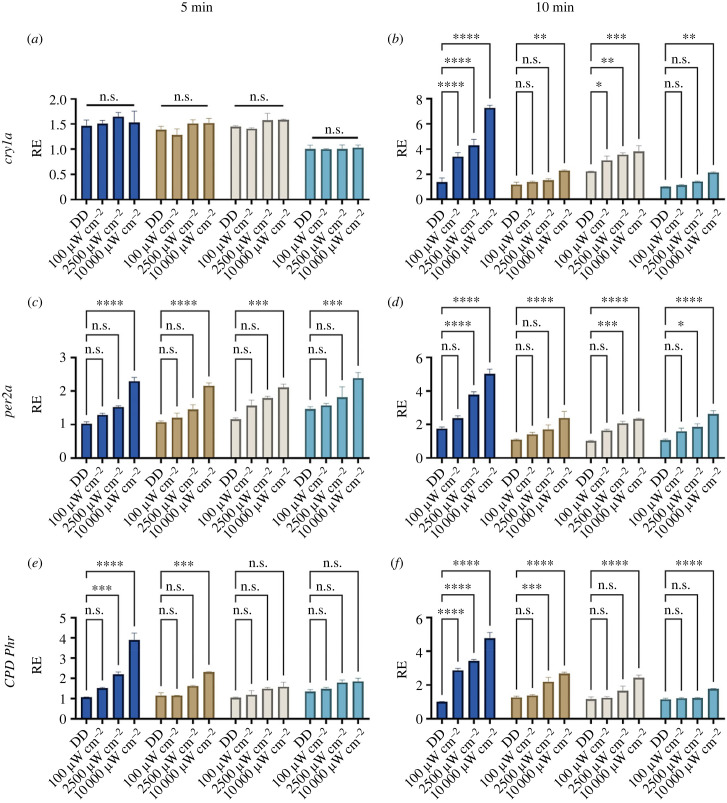
Induction of light-inducible genes in cavefish cell lines. Cell lines were subjected to the light of three different intensities as well as a non-light pulsed dark control for 5 min and 10 min. The different colours denote the different cell culture from the different cave populations; dark blue = surface, brown = Pachón, beige = Chica, teal = Tinaja. RE of genes was determined by RT-qPCR for (*a*) 5 min *cry1a,* (*b*) 10 min *cry1a,* (*c*) 5 min *per2a*, (*d*) 10 min *per2a*, (*e*) 5 min *CPD*-*Ph* and (*f*) 10 min *CPD*-*Ph*. RE is plotted against lowest expressed gene across strains. Normalized against housekeeping gene RLP-13*α*. A two-way ANOVA with a Tukey post-test was used to determine significance (*n* = 3–4). **** = *p* < 0.0001, *** = *p* < 0.001, ** = *p* < 0.01, * = *p* < 0.05, n.s. = not significant.

The 5 min intensity response curves showed similar responses across all strains (figure 1*a,c,e*). We do not observe any induction of *cry1a* in response to a 5 min light pulse in any of the strains, but both *per2a* and *CPD-Ph* is induced significantly at 10 000 µW m^−2^ creating an approximately 1.7-fold induction across the strains for both genes (figure 1). The differences between the strains are more striking when looking at the 10 min light pulses. Not surprisingly, surface fish cells are by far the most light responsive, with significant induction of all genes at all intensities explored. This is particularly evident for *cry1a* which is induced 5.6-fold in surface cells at 10 000 µW m^−2^ while the same gene is only induced by 1.7- to 1.9-fold in the cells from the cave strains. In fact, the lowest intensity of 100 µW m^−2^ induces *cry1a* 2.6-fold in surface cells, giving a larger response than any of the cave cells at 10 000 µW m^−2^ (figure 1*b*). *CPD-Ph* is induced by a 5 min light pulse by the higher intensity light in surface cells, while only Pachón has a small response to 5 min 10 000 µW m^−2^ (figure 1*e*). All intensities induce *CPD-Phr* in surface cells, with a fivefold induction in surface cells compared to 2- to 3.6-fold induction in the cave cell lines (figure 1*f*).

### (b) Blind cavefish cells have an entrainable circadian clock

After establishing that the embryonic cell lines are indeed light responsive, we wanted to examine if they are entrainable and have a circadian clock. Cells were seeded as described above and kept on a 12 : 12 LD cycle for 3 days, before starting sampling on the fourth day, at 6 h intervals, starting at ZT3 on the LD cycle. On the following day, the remaining cells were transferred to a dark–dark (DD) regime and harvested at 6 h intervals starting with CT3.

**Figure 2.**
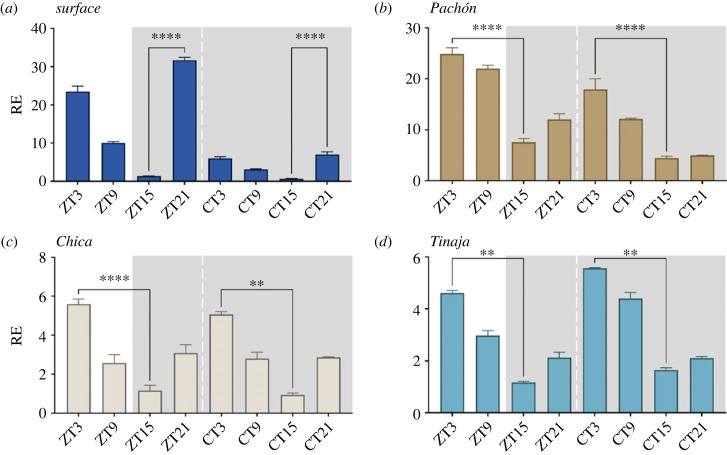
*per1* rhythmic expression in cavefish cell lines. Cell lines were sampled every 6 h on a light–dark cycle and then subsequently on a free-running cycle. Light area represents the day, while dark grey area represents the dark (12 : 12 LD cycle). ZT = zeitgeber. The different colours denote the different cell culture from the different cave populations; dark blue = surface, brown = Pachón, beige = Chica, teal = Tinaja. RE of *per1* was determined by RT-qPCR (*a*) surface cells, (*b*) Pachón, (*c*) Chica and (*d*) Tinaja. RE is plotted against lowest expressed timepoint across all strains. A two-way ANOVA with a Tukey post-test was used to determine the significance between peaks and troughs (*n* = 3–4). **** = *p* < 0.0001, *** = *p* < 0.001, ** = *p* < 0.01. Normalized against housekeeping gene RLP-13*α*. RAIN, a non-parametric rhythmicity test [40] was used to determine significance and period length in dataset electronic supplementary material, file S1.

All cell lines show both entrained and free-running rhythmic expression of *per1* (figure 2).

Surface cells oscillate strongly on an LD cycle with a peak expression at ZT3 and through at ZT9 (19-fold difference), matching that of the adult fish and embryos [19,26]. The oscillations persist in DD but are considerably dampened to just a sixfold difference, after just 1 day in constant darkness (figure 2*a*). The cave strains also entrain to an LD cycle with free-running rhythms in DD, all with peaks at ZT/CT3, but with troughs at ZT/CT15 instead of ZT/CT9 for surface fish. The amplitude of *per1* is reduced in cave cells, with an approximately 4- and approximately 4.5-fold difference for Pachón and Chica respectively, compared to just an approximately threefold difference for Tinaja (figure 2*b–d*).

### (c) Cavefish cell lines show raised basal levels of light-inducible genes *per2a* and *CPD-Phr*

**Figure 3.**
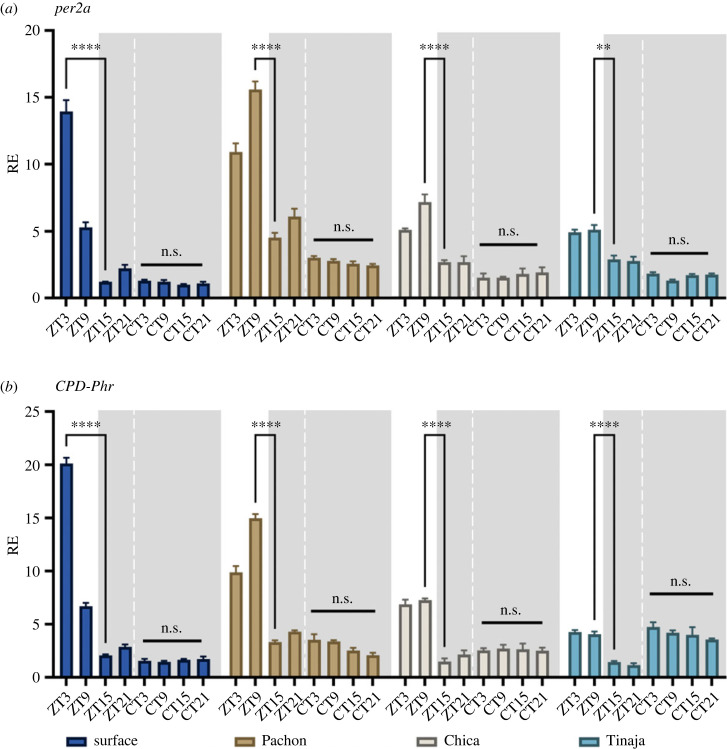
*per2a* and *CPD-Phr* expression in cavefish cell lines in LD and DD. Cell lines were sampled every 6 h on a light–dark cycle and then subsequently on a free-running cycle. Light area represents the day, while dark grey area represents the dark (12 : 12 LD cycle). ZT = zeitgeber. The different colours denote the different cell culture from the different cave populations; dark blue = surface, brown = Pachón, beige = Chica, teal = Tinaja. RE was determined by RT-qPCR (*a*) *per2a* and (*b*) *CPD-Phr*. RE is plotted against lowest expressed gene. Normalized against housekeeping gene RLP-13*α*. A two-way ANOVA with a Tukey post-test was used to determine significance between peaks and troughs (*n* = 3–4). **** = *p* < 0.0001, ** = *p* < 0.01, n.s. = not significant.

Using the same entrained and free-running dataset, we also examined the same light-inducible genes, *per2a* and *CPD-Phr* expression to see if they follow the same patterns as we have reported for early development and adult data. Both genes are expressed during the light phase across all cell cultures with no significant induction in the dark phase (figure 3). While acrophase of *per2a* and *CPD-Phr* is at ZT3 in surface cells, per2a and CPD expression is altered and delayed in the cave strains, with Pachón showing a peak expression at ZT9 for both genes, while Chica and Tinaja have a less defined peak of expression for both genes (figure 3). The fold response is highest in surface, with 12-fold induction of *per2a* and 10-fold induction of *CPD-Phr*. While Pachón has a higher absolute expression, than Chica and Tinaja, the relative fold change between peak and trough expression is significantly lower across the cave strains with 3.5-fold for Pachón, 2.7-fold for Chica and 1.8-fold differenece for Tinaja. *CPD-Phr* fold change is reduced but similar across strains with Pachón, Chica and Tinaja showing a 4.5, 4.9 and 3.7-fold change, respectively. Basal transcription of both genes is increased in DD in the cave strains.

### (d) Embryonic response

We have previously published on the beginning of light responsiveness and the start of the circadian clock in the developing surface versus Pachón cavefish embryo [26]. In that study, we found that surface embryos were earlier to develop responsiveness to light and their clock started ‘ticking’ slightly earlier than Pachón cavefish embryos. As we have made cell lines from two additional cavefish, Chica and Tinaja, but do not have matching rhythmic qPCR data from Chica or Tinaja, we decided to replicate the experiment for developing Chica and Tinaja embryos.

**Figure 4.**
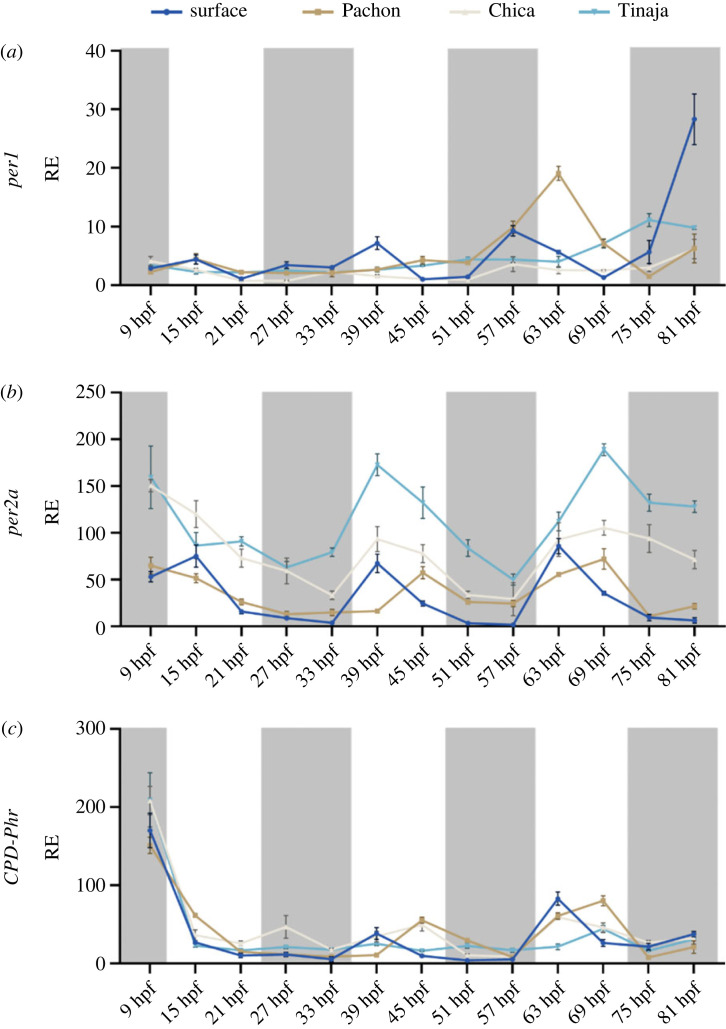
Early embryonic expression of key clock genes in developing cavefish embryos on a light:dark cycle. Developing embryos were sampled from 9 hpf every 6 h on a light–dark cycle until 81 hpf. Light area represents the day, while dark grey area represents the dark (12 : 12 LD cycle). The different colours denote the different cell culture from the different cave populations; dark blue = surface, brown = Pachón, beige = Chica, teal = Tinaja. RE was determined by RT-qPCR (*a*) *per1*, (*b*) *per2a*, (*c*) *CPD-Phr*. RE is plotted against lowest expressed gene. Normalized against housekeeping gene RLP-13*α*. Associated statistics can be found in the electronic supplementary material, file S2. Data on surface and Pachón were published in 2018 [26].

Chica and Tinaja embryos were collected from various mating pairs and raised either on an LD or DD cycle in constant temperature. Embryos were collected every 6 hours from 9 hours post fertilization (hpf) to 81 hpf. While surface and Pachón starts showing cycling *per1* transcript on the third day of development, neither Chica nor Tinaja embryos show any clear rhythmic pattern by 81 hpf (figure 4*a*). They are, however, both light responsive on the second day, although the acrophase changes on the subsequent cycle, while this is not the case for surface embryos that retain the same *per2a* expression pattern throughout development (figure 4*b*). *CPD-Phr* expression patterns are much less robust in Chica and Tinaja, with possible weak induction around 63–69 hpf, however, it is difficult to determine if this is real or if it is due to the increase of transcriptional activity around this time of development (figure 4*c*).

**Figure 5.**
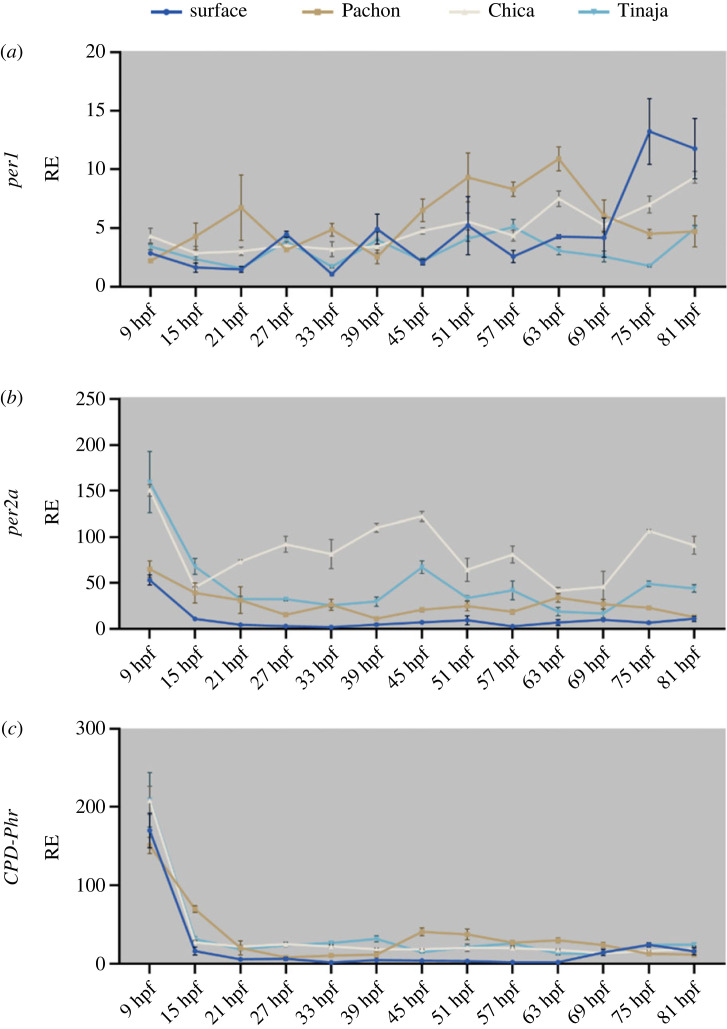
Early embryonic expression of key clock genes in developing cavefish embryos in complete darkness. Developing embryos were raised in complete darkness and sampled every 6 h from 9 hpf until 81 hpf. The different colours denote the different cell culture from the different cave populations; dark blue = surface, brown = Pachón, beige = Chica, teal = Tinaja. RE was determined by RT-qPCR (*a*) *per1*, (*b*) *per2a* and (*c*) *CPD-Phr*. Relative expression is plotted against lowest expressed gene. Normalized against housekeeping gene RLP-13*α*. Associated statistics can be found in the electronic supplementary material, file S2. Data on surface and Pachón were published in 2018 [26].

Embryos that are raised in complete darkness do not exhibit any circadian rhythms, which is in line with zebrafish findings, meaning that embryos do not seem to inherit a ‘maternal rhythm’ (figure 5*a*). The light-inducible genes *per2a* and *CPD-Phr*, both show a higher basal expression across cave strains against surface embryos with no apparent cycling (figure 5*b,c*). It is also worth noting that all embryos have what is likely to be a high amount of maternally deposited *per2a* and *CPD-Phr*, which is quickly depleted during the first 15 h of development (figures 4*b,c* and 5*b,c*).

## 3. Discussion

In this paper, we have shown that despite cavefish being blind, they still have cells that can detect light, entrain cellular clocks on an L–D cycle, as well as having an endogenous circadian rhythm in free-running conditions. Non-visual light detection is retained at a fundamental cell-based level. These embryonic cultures are easy and cheap to make and maintain and have great potential as a tool not only for circadian research but are also particularly suitable for experiments that require multiple experimental conditions and numerous replicates that require a lot of biological material.

### (a) Acute light sensitivity is altered in cave cell lines compared to surface fish

Here, we have shown that just a short light pulse can induce light-responsive genes in both cave and surface cells, with the latter being the most light responsive both in terms of intensity, duration and fold induction. Though light sensitivity is retained at a cellular level, there is a strong reduction in this responsiveness within the cave populations. The differences in this response between the Pachón, Chica and Tinaja cave strains is, however, more subtle. Not surprisingly, both duration and intensity of light pulse has an impact on the fold induction of light-sensitive genes. This is particularly evident for *cry1a* that shows no light induction in response to a 5 min light pulse regardless of intensity, while in surface cells both *CPD-Phr* and *per2a* are induced by a 5 min-high intensity light pulse. All cave cells do respond to light across all genes explored, but their fold response is dampened and require high intensity light. The difference between the light response for various genes might reflect differences in the light-input pathways for these genes. Data from zebrafish shows that the light induction of *cry1a* requires active protein synthesis and most likely works through a D-box element within its promoter. Whereas *per2* induction does not require de novo protein synthesis and is more E-box dependent [39]. The difference in response we see in cavefish cells between 5 and 10 min light pulses might reflect a similar difference in signalling pathways, with *cry1a* requiring novel protein synthesis and a longer duration of light stimulation before an increase of transcript is detectable. It is even possible that different photopigments might act to stimulate these differential input pathways, creating even greater cellular flexibility. It is likely that evolutionary change in the cave could be different for alternate signalling routes from light, as well as promoter changes that regulate rhythmic or basal gene expression levels. These new cavefish cell lines might finally offer up a tool with which to explore such evolutionary changes more easily at a cellular level.

We should, however, also keep in mind that the peak phase of light sensitivity may be altered in cave cell lines, or even adult fish for that matter. Data from adult fin clips [19], embryo [26], as well as the cell cultures, suggest that peak expression of light-induced genes on an LD cycle is altered in cavefish. Experiments performed here may therefore miss the peak light sensitivity of the cavefish due to changes in the timing of the molecular clock mechanism. Simply put, is ZT21 in the clock mechanism molecularly the same between surface and cave populations? Phase response curves will need to be done for all cavefish and surface fish to determine potential differences in peak light sensitivity, an experiment that is suited for transgenic cell lines. Creating transgenic cell lines can be challenging in fish cells, as in many other cell types, in part due to transfection efficiencies. Nevertheless, this has been successfully achieved by numerous groups in differing fish species cell cultures and has created some invaluable tools for the study of cell function [36,41,42]. Last year, Krishnan *et al*. [43] reported that transgenic cavefish liver cell lines can be generated with relative ease by electroporation. Luciferase reporter lines have proven a very useful tool in zebrafish [44] and are likely to be a useful tool in cavefish. Furthermore, such reporter cell lines may also reveal more detailed differences among the different cave strains with a higher resolution dataset. As such, our new, cavefish cell cultures provide an excellent basis for the future development of transgenic reporter cell lines and even cell-based CRISPR-Cas9 approaches.

### (b) Entrainment in cell lines mimics that of adult fish

All cells entrain on an LD cycle, and rhythms persist in DD. The amplitude is however quite reduced in comparison to adult fin clips, which is particularly evident under free-running conditions. There is no easily apparent explanation for this other than possible differences in clock function between cell-types, or the generally greater robustness of clocks when examined *in vivo*. This contrasts with zebrafish cell lines, however, that have high-amplitude rhythms that persist with the same period but dampening in amplitude in free-running conditions over several days [44]. The cell lines do however share the acrophase of *per1* with adult fin clip data [19], where surface fish peak at ZT21 and cavefish cells are delayed to ZT3*.* In many respects, this is very interesting, as the fundamental differences in core clock timing previously reported from adult samples are retained at the cell culture level. *Per2a* and *CPD-Phr* expression patterns in cave cells also have an altered acrophase to surface cells, as well as a reduced fold change. We also observe this in the embryo data from the third day of development, yet this change in acrophase is not observed in Chica and Pachón *per2a* fin clips data (no data exist yet for Tinaja), but the widening of the peak *CPD-Phr* expression is, however, observed in adult fin clip data, though the exact expression profiles do not match [19]. It is possible that this may be due to variations in light intensities used for entrainment between the experiments. Another possibility is that there is a phase difference between organs; however, this has never been explored experimentally in cavefish. There is, however, evidence supporting this from a study on the flatfish *Scophtalamus maximus* where organs are reported to have slightly different acrophases across clock genes such as *per2*, *per1* and *cry1* [45].

### (c) Variations in expression suggest light input and clock have evolved independently in different cave strains in response to an arrhythmic environment

By using four different genes, three clock genes: *per1, per2a* and *cry1a* as well as one photolyase, paired with acute light-pulsing experiments and expression across circadian cycles, we can capture nuances not previously explored in the light responses across cave cells. The three different cave strains are geographically isolated from each other, meaning that they have evolved independently to an environment without zeitgebers. Although we see similarities across these cave strains, such as increased basal transcript of *per2a* and *CPD-Phr* and changes in acrophase, there are also interesting differences. The embryonic data suggest that the different strains develop light responses quite early on, but that the Pachón clock is the earliest to ‘start ticking’. Furthermore, Pachón shows a clear change in peak expression of *per2a* and *CPD-Phr* on an LD cycle, compared to Chica and Tinaja. Tinaja is the strain that has the lowest relative change in response to light in the genes explored. These findings are in line with previous reports [11,19,26], including a more recent data on circadian transcriptomes of cavefish, that show great variation in the molecular ‘disruptions’ seen following evolution in the dark [46]. These data underpin the hypothesis that disruptions (mutations in) to the biological clock have evolved independently, and through different molecular mechanisms in different populations of cavefish.

In this article, we have shown that that cell cultures from cavefish can be easily generated and maintained. We have provided proof that despite being blind, cells from the Mexican blind cavefish can detect light and entrain their clocks to a LD cycle differently to that of terrestrial surface fish. Cell lines presented here, as well as cell lines created from *A. mexicanus* liver cells [43] as well as the EPA cell line created from the Somalian cavefish *P. andruzzii* [24] is proof that cell cultures are a valuable tool in the study of adaptation to an environment in the dark.

## 4. Material and methods

### (a) Biological materials and embryo maintenance

Adult surface, Pachón, Tinaja and Chica cavefish were maintained at 22–25°C and exposed to a 14 : 10 h photoperiod. Four–six pairs of fish were mated every 3–4 weeks by *in vitro* fertilization. Embryos were transferred to E3 fish water (5 mM NaCl, 0.17 mM KCl, 0.33 mM CaCl2, 0.33 mM MgSO4, 0.00001% Methylene Blue) with 20 embryos per 25 ml flasks, making up one biological replicate. When handling embryos, we recommend using clean but previously used culture plastics and stripettes (or glass) as the chorion of *A. mexicanus* tends to stick to new plastic. The embryos were kept at a constant 25°C and placed on a 12 : 12 DL cycle or in constant darkness within 1–2 h of fertilization. Twenty embryos were sacrificed from 9 hpf and then at 6 h intervals in TRIzol Reagent (Invitrogen), homogenized and stored at −20°C. All animals were maintained in a Home Office approved facility and handled in accordance with the Animal Welfare Act of 2006.

### (b) Embryo dissociation and creation of *Astyanax mexicanus* cell lines

Embryos were collected and maintained as above until 24–30 hpf after the cavefish have hatched. In a tissue culture hood, around 50 newly hatched embryos per strain were washed five times in a 2 ml Eppendorf tube with 1.5 ml 1× PBS per wash. A new tube was used per wash to minimize the chance of contamination. Embryos were then dissociated in 1 ml of 0.5% Trypsin—no phenol red (Gibco) for 20 min at room temperature (RT), pipetting vigorously up and down with a P1000 and P200 every 3 min to help break up the embryos. Dissociated cells were transferred to a 25 cm^2^ flask (Greiner) with 7 ml of fish cell culture media (Leibovitz −15 medium (Gibco) supplemented with 15% FBS (Biowest), 0.05 mg ml^−1^ of gentamicin (Gibco) and 1 × penicillin-streptomycin (Gibco)) and kept in a cell culture incubator at 28°C, no CO_2_. The following day, most cells had adhered, culture media was changed and any debris or cells that had not adhered to the plastic were discarded. Primary culture confluency was reached after approximately two weeks. Upon reaching confluency, cells were washed with 1× PBS at RT. The *Astyanax mexicanus* cell cultures are very adherent compared to zebrafish PAC2 cell lines, so incubation with 2 ml of 0.5% Trypsin for 10 min at RT would typically be used to dissociate cells from plastic in a small 25 cm^2^ flask. Cells were split 1 : 7 every 5–7 days for two months before experiments started. All four cave variant cell cultures can be frozen at −80°C in freezing media (L-15 (Gibco), 30% FBS (Biowest) 10% DMSO (ThermoFisher)). See electronic supplementary material, figure S1 for photos of cell morphology.

### (c) Intensity response curves and circadian experiments in cell lines

For the intensity response curve experiments, 150 000 cells were seeded in 3 cm dishes (Nunc), and for the circadian experiments, the same number of cells were seeded in six-well plates (Nunc). The cell seeding took place around mid-day, and the cells were entrained for three complete 12 : 12 LD cycles at 28°C before experiments and sampling started on the fourth day, at this time cells were confluent. For the circadian experiments, entrained cells were sampled at ZT3, ZT9, ZT15 and ZT21, while free-running cells were sampled at CT3, CT9, CT15 and CT21. Cells were washed with 1 × PBS before being homogenized in 1 ml of TRIZol using a cell scraper and stored at −20°C. For the light-pulsing experiments, cells were pulsed using a halogen light source and fibre optic (Cairn Research UK), filtered to 400–700 nm with LOT-Oriel filters (Newport Corporation) for 5 or 10 min, at three different intensities 100 µW cm^−2^, 2500 µW cm^−2^ and 10 000 µW cm^−2^, measured with a Macam power meter (now sold as Irradian PM203). The light-box has a temperature-controlled chamber that ensures temperatures are the same across all light intensities. After the light pulse, cells were kept in the dark at 28°C for 3 h to allow for transcription of light-induced genes, before being sacrificed in 1 ml of TRIzol, as described above. Dark controls were also harvested at this time.

### (d) RNA, cDNA and RT-qPCR

RNA was extracted from homogenized cells, tissues or embryos according to the manufacturer's guidelines (TRIzol, Invitrogen). RNA concentration was determined by NanoDrop2000 Spectrophotometer (Thermofisher) and integrity was determined by running 1 µl of RNA and 9 µl 11.1% glycerol on EtBr 1.5% agarose gel in 1 × TAE. cDNA was synthesized from 2 μg of RNA 2 µg of RNA was reverse transcribed to cDNA using Superscript II Reverse Transcriptase with random hexamers and oligo dT primers (all from Invitrogen).

RT-qPCR was performed on a C1000 Touch Thermal Cycler with the CFX96 Optical Reaction Module (Bio-Rad) using 2× KAPA SYBR FAST qPCR mix (Kapa Biosystems) in technical triplicates with gene specific-primers, see [19,26], following cycling temperatures and times as per manufacturers protocol. ΔCt was determined using reference genes using rpl13*α* or EF1*α* as a reference gene and relative expression (RE) levels were compared using the ΔΔCt method.

### (e) Statistical analysis

The data in this study are presented as the mean ± s.e. of the mean (*n* = 3–4). Statistical packages from GraphPad Prism 9 were used to determine significance. Rhythmicity tests were performed using the RAIN (Rhythmicity Analysis Incorporating Non-parametric Methods) statistical package of R, with default settings [40]. Peak-troughs of rhythmic data were also assessed with Tukey post-test. All stats can be found in electronic supplementary material, Excel file S1 and S2.

## Data Availability

RT-qPCR data can be found in the electronic supplementary material, Excel files S1 and S2 under the Data tab [47].
